# Case report: Absent fluorodeoxyglucose uptake in a dog with unexpected brain death

**DOI:** 10.3389/fvets.2022.902475

**Published:** 2022-12-01

**Authors:** Yoonhoi Koo, Yejin Na, Taesik Yun, Yeon Chae, Dohee Lee, Hakhyun Kim, Mhan-Pyo Yang, Byeong-Teck Kang

**Affiliations:** Laboratory of Veterinary Internal Medicine, College of Veterinary Medicine, Chungbuk National University, Cheongju, South Korea

**Keywords:** brain death, coma, positron emission tomography, nuclear scan, dog

## Abstract

A 5-year-old male Maltese dog was presented with generalized tonic seizures and hypermetria. Multiple nodular subcortical cerebellar enhancements and meningeal enhancement were observed on magnetic resonance imaging. Fluorodeoxyglucose-positron emission tomography/computed tomography was performed due to suspicion of neoplastic disease, and no fluorodeoxyglucose uptake was observed in the intracranial structures. In PET images of this dog, absent fluorodeoxyglucose uptake was identified in the brain indicating no cerebral metabolism, strongly suggested brain death. The dog had no spontaneous breathing and no brainstem reflexes for more than 24 h after the termination of anesthesia. Through these results, this dog was diagnosed with unexpected brain death, and it is presumed that the cause was anesthesia. We report herein a case of brain death in a dog diagnosed using fluorodeoxyglucose-positron emission tomography/computed tomography.

## Introduction

Brain death is defined as the irreversible and complete loss of all activities of the brain, brainstem, and cerebellum resulting from the cessation of cerebral perfusion caused by increased intracranial pressure (1). The diagnosis of brain death is primarily clinical; that is, it involves a single apnea test and two assessments of brainstem reflexes (2). Exclusion criteria for the diagnosis of brain death include hypothermia, shock, brainstem encephalitis, Guillain-Barre syndrome, encephalopathy associated with hepatic failure, uremia and hyperosmolar coma, severe hypophosphatemia, and drugs known to alter neurologic and neuromuscular function and electroencephalographic testing (2, 3). Brain death can be diagnosed through ancillary tests when a clinical examination is not fully possible (4). Electroencephalography, cerebral angiography, nuclear scan, transcranial Doppler, computed tomography angiography, and magnetic resonance angiography are ancillary tests currently used to determine brain death (4, 5).

Human patients diagnosed with brain death using a fluorodeoxyglucose-positron emission tomography (FDG-PET) scan, which is a nuclear scan, have been reported (1, 6, 7). A hallmark of the FDG-PET scan for brain death is the absence of tracer residues within the brain and brainstem, indicating the absence of brain metabolism (1, 6, 7). Furthermore, in a study using brain-dead experimental animals, FDG-PET was used to confirm brain death (8).

To the author's knowledge, there have been neither reports of brain death in canine patients in clinical veterinary medicine, nor of canine patients diagnosed with brain death using FDG-PET.

## Case description

**Figure 1:**
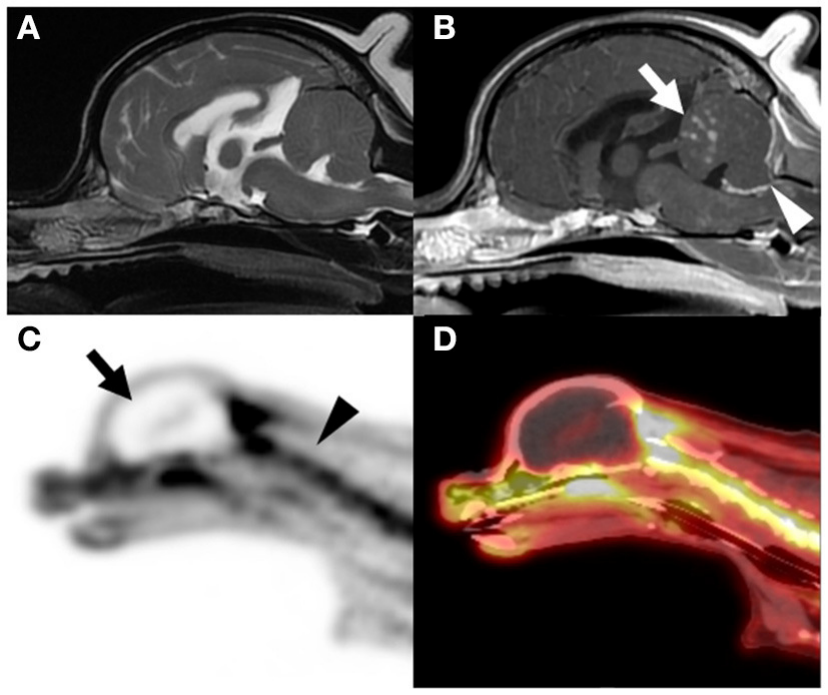
Magnetic resonance imaging (MRI) and positron emission tomography/computed tomography (PET/CT) scans of the brain. Intracranial lesion was not observed on the T2-weighted MRI scans **(A)**. However, multiple nodular subcortical cerebellar enhancement [arrow in **(B)**] and meningeal enhancement [arrowhead in **(B)**] were observed on the contrast-enhanced T1-weighted MRI scan **(B)**. Three-dimensional projection images of the fluorodeoxyglucose (FDG) uptake in a sagittal PET scan **(C)** and sagittal PET/CT scan **(D)** are shown. Absence of FDG uptake was indicative of cessation of the glucose metabolism in the brain tissue [arrow in **(C)**] when compared with the presence of FDG uptake in the cervical spinal cord [arrowhead in **(C)**], as observed in the PET/CT scans. A hollow skull or empty light bulb sign, a typical PET image of a brain-dead patient, was observed [arrow in **(C)**].

A 5-year-old male Maltese dog was admitted with hypermetria that started 3 days prior and generalized tonic cluster seizures lasting <3 min. It was confirmed through the history taking that the dog had not been admitted to other veterinary clinics for the neurologic symptom, and that this dog had never been under general anesthetized. Hypermetria of both thoracic limbs as cerebellar ataxia was identified through neurological examination. Abnormal findings were not identified in the blood test, and it was confirmed that general anesthesia was acceptable for the canine patient (Table 1). Multiple nodular subcortical cerebellar enhancements and meningeal enhancements in the cerebellum and cervical spinal cord were observed on contrast magnetic resonance imaging (MRI; 1.5-T magnet, SIGNA Creator; GE Healthcare, Milwaukee, WI, USA) (Figures 1A,B). Although this MRI pattern has never been reported in dogs, this MRI pattern has been reported as a metastatic lesion in humans (9); therefore, screening for primary tumor and evaluation of cerebellar lesion in this case was required.

**Table 1:** Blood analysis results before anesthesia.

	**Before anesthesia**	**Reference range**
WBC (10^3^/μL)	8.77	5.05–16.76
Hemoglobin (g/dL)	13.3	13.1–20.5
Platelet (10^3^/μL)	462	148–484
Total protein (g/dL)	6.8	5.4–7.1
Albumin (g/dL)	4.0	2.6–3.3
ALT (IU/L)	43	21–102
AST (IU/L)	14	23–66
ALP (IU/L)	99	29–97
Urea nitrogen (mg/dL)	13.4	7–25
Creatinine (mg/dL)	0.5	0.5–1.5
Sodium (mmol/L)	152	141–152
Potassium (mmol/L)	5.0	3.6–5.8
Chloride (mmol/L)	115	105–115
Calcium (mg/dL)	10.3	9–11.3
Phosphate (mg/dL)	3.3	2.6–6.2

PET/computed tomography (PET/CT; Discovery-72 STE, General Electric Medical Systems, Waukesha, WI, 73 USA) was performed immediately after MRI to screen for primary or metastatic brain tumors. PET/CT images were obtained approximately 60 min after intravenous administration of 6.3 MBq/kg fluorodeoxyglucose (FDG). The PET/CT images showed an absence of FDG uptake in the intracranial structures (Figures 1C,D). To investigate the metabolism in the brain, standardized uptake values (SUVs), which is used to normalize tissue FDG concentration relative to the injected dose and body weight, were compared with other extracranial structures. The estimated mean SUVs of the cerebrum (0.09), brainstem (0.27), and cerebellum (0.15) were lower than those of the cervical spinal cord (2.42), trapezius muscle (1.01), and both eyes (1.24). With the exception of intracranial structures, FDG uptake was identified in the whole body, and indications of a neoplastic lesion were not observed.

For MRI and PET/CT scans, general anesthesia was performed for a total of 3 h, and major vital signs were measured at 5-min intervals. The ranges of the vital signs that were monitored during anesthesia were as follows: heart rate, 90–120; respiratory rate, 12; body temperature, 36.5–38.1; systolic blood pressure, 105–145; end-tidal carbon dioxide, 35–44; saturation of percutaneous oxygen, 97–100. Following anesthesia termination, the canine patient was found to be in an irreversible coma, with the absence of all cranial nerve reflexes, including pupillary light reflex, oculocephalic reflex, corneal reflex, facial muscle movement to noxious stimulus, and pharyngeal reflex. In the absence of spontaneous respiration, mechanical ventilation was maintained. The canine patient remained in an irreversible coma for 24 h. The next day, at the request of the owner, the canine patient was euthanized, and we did not perform a post-mortem examination.

## Discussion

Brain death in human patients accounts for approximately 5% of pediatric and 2% of adult in-hospital deaths in the United States (4). However, to the author's knowledge, there have been no reports of brain death in canine patients due to diagnostic limitations. Scintigraphy is an inexpensive and non-invasive examination used to determine brain death in human patients (1, 5). Ozdemir suggested that FDG-PET/CT could be used for the confirmation of brain death because it has higher spatial resolution compared to other nuclear scans, such as gamma camera and single-photon emission computed tomography (1). In this comatose canine patient without spontaneous breathing, we suspected brain death due to the absence of brainstem reflexes including the pupillary light reflex, oculocephalic reflex, corneal reflex, facial muscle movement to noxious stimulus, and pharyngeal and tracheal reflex. The absence of brainstem reflexes is considered to be one of the diagnostic criteria for brain death in human patients (4, 5). In addition, the absence of FDG uptake in intracranial structures, an indicator also used on the FDG-PET scans of brain-dead human patients, was identified in this canine patient (1, 6, 7). A previous study reported that extremely low FDG uptake was identified in the brain in human patients with metastatic neuroblastomas; however, no neurological symptoms were observed in those patients (10). In our case, since this canine patient showed an absence of all brainstem reflexes, the possibility of neuroblastoma was considered to be very low.

It is suggested that a 6-h observation period after the first clinical examination is reasonable and sufficient for clinical manifestations inconsistent with the diagnosis of brain death (2). After the canine patient was confirmed as brain dead, a neurologic examination was performed every 12 h for 24 h, and the results of the second examination were the same as those of the first. Therefore, this canine patient was determined to be in an irreversible coma.

As histopathology was not performed in this case, the disease resulting in the distinct lesion observed on the MRI scan could not be identified. Although an obvious lesion was not observed on the MRI scan, brainstem encephalitis could not be completely excluded. It has been reported that in the cases of human brainstem encephalitis such as Fisher syndrome, increased FDG uptake in the lesions were identified on PET scans (11). Therefore, the possibility of brainstem encephalitis, which was one of the exclusion criteria for the determination of brain death, was excluded in this case because FDG uptake was absent in the brain of this canine patient (2, 3). Other exclusion criteria, including hypothermia, shock, encephalopathy with hepatic failure, uremia and hyperosmolar coma, and severe hypophosphatemia, were excluded in this case due to normal results of the physical examination and blood analysis before the anesthesia (2, 3). Furthermore, changes in vital signs and saturation of percutaneous oxygen and end-tidal carbon dioxide were not observed during or after anesthesia. Therefore, brain death was confirmed in this canine patient.

It has been shown that the complication rate due to anesthesia is higher in canine patients with brain lesions than in canine patients without brain lesions due to the increase in intracranial pressure following anesthesia (12). Several reports have suggested that brain death can occur during anesthesia (13, 14). The exact cause of brain death in this canine patient was not identified; however, we suspected that the cause was an anesthesia-induced increase in intracranial pressure followed by brain damage.

This report has several limitations. First, the apnea test, one of the tests used to confirm brain death, was not performed in this canine patient due to failure of arterial catheter placement. Second, other ancillary tests used to determine brain death, including electroencephalography, cerebral angiography, transcranial Doppler, computed tomography angiography, magnetic resonance angiography, and brainstem auditory evoked response, were not performed. Third, intracranial disease, which may have been related to brain death in this canine patient, was not diagnosed through histopathology.

In this case, unexpected brain death was confirmed in the canine patient through the absence of all cranial nerve reflexes, as well as the absence of FDG uptake in the brain, a marker for the absence of brain metabolism. This report described the diagnosis of brain death in a canine patient for the first time in clinical veterinary medicine, and described the features of FDG-PET/CT of brain death in a canine patient.

## Data availability statement

The raw data supporting the conclusions of this article will be made available by the authors, without undue reservation.

## Ethics statement

Ethical review and approval was not required for the animal study because this is a case report. Written informed consent was obtained from the owners for the participation of their animals in this study.

## Author contributions

YK, YN, TY, and B-TK contributed to management of the case. YK and TY were in charge of the PET/CT evaluation. YK wrote the first draft of the manuscript. YK, YC, DL, HK, M-PY, and B-TK participated in the revision of the manuscript. All authors read, commented on, and approved the final manuscript.

## Funding

This work was supported by the National Research Foundation of Korea (NRF) grant funded by the Korean Government (MSIT), number 2021R1A2C1012058, and by Korea Institute of Planning and Evaluation for Technology in Food, Agriculture, Forestry (IPET) through Companion Animal Life Cycle Industry Technology Development Program, funded by Ministry of Agriculture, Food and Rural Affairs (MAFRA) (322095-04).

## Conflict of interest

The authors declare that the research was conducted in the absence of any commercial or financial relationships that could be construed as a potential conflict of interest.

## Publisher's note

All claims expressed in this article are solely those of the authors and do not necessarily represent those of their affiliated organizations, or those of the publisher, the editors and the reviewers. Any product that may be evaluated in this article, or claim that may be made by its manufacturer, is not guaranteed or endorsed by the publisher.
